# A84 FACTORS ASSOCIATED WITH HIGH LEVELS OF TELEPHONE CARE SATISFACTION AMONG INDIVIDUALS WITH INFLAMMATORY BOWEL DISEASE

**DOI:** 10.1093/jcag/gwac036.084

**Published:** 2023-03-07

**Authors:** J N Foncham, N Rohatinsky, S Fowler, J N Peña-Sánchez

**Affiliations:** 1 Community Health and Epidemiology; 2 College of Nursing; 3 College of Medicine, University osf Saskatchewan, Saskatoon, Canada

## Abstract

**Background:**

Individuals with inflammatory bowel disease (IBD) require regular medical follow-up with gastroenterology care providers. Individuals in rural areas face barriers in assessing specialized IBD care. Virtual care (VC) may act as a solution. The coronavirus disease 2019 pandemic increased the use of VC, particularly telephone care (TC) appointments in Saskatchewan, Canada. There is limited evidence around the levels and factors associated with satisfaction with TC among individuals with IBD.

**Purpose:**

This study aims to measure satisfaction with TC in individuals with IBD who live in Saskatchewan, Canada, and evaluate the factors associated with TC satisfaction.

**Method:**

A cross-sectional study was conducted among individuals with IBD through an online survey between December 2021 and April 2022 in Saskatchewan. The Telephone Care Satisfaction Questionnaire for individuals with IBD (TCSQ-patient) was a 16-item questionnaire used to measure TC satisfaction on a scale from 1 (very dissatisfied) to 7 (very satisfied). The online survey also included the Quality of Care Through the Patient’s Eyes-IBD (QUOTE-IBD) questionnaire, Short Inflammatory bowel disease questionnaire (SIBDQ), and demographic questions. Factors associated with TC satisfaction were explored using linear regression models. A backward model building strategy was used, and 95% confidence intervals (95%CI) were reported.

**Result(s):**

In total, 87 individuals with IBD participated in the study. Among the study participants, 54 (64.3%) had Crohn's disease, 53 (61.6%) were female, 60 (69.8%) lived in urban centres, and 37 (43.5%) were between 41-59 years old. The mean satisfaction with TC was 5.70 (SD=0.94). In addition, the means of the QUOTE-IBD and SIBDQ were, respectively, 8.96 (SD=1.70) and 48.14 (SD=13.02). In the bivariate analysis, area of residence (rural vs. urban) and health related quality of life quality (SIBDQ>50 vs SIBDQ<50) were associated with satisfaction with TC, respectively, 0.47 (95%CI 0.02-0.91) and 0.48 (95%CI 0.08-0.88). Adjusting by gender, age group, type of disease, and health care provider managing IBD, we identified that the satisfaction with TC was 0.48 (95%CI 0.02-0.94) higher among individuals with IBD living in rural Saskatchewan in comparison to their urban counterparts.

**Conclusion(s):**

Individuals living with IBD in Saskatchewan reported high levels of satisfaction with TC. Rural residence is associated with higher levels of TC satisfaction. These results could help in the promotion of TC utilization and improve access to specialized IBD care, especially among those living in rural areas.

**Please acknowledge all funding agencies by checking the applicable boxes below:**

Other

**Please indicate your source of funding;:**

Saskatchewan Health Research Foundation (SHRF)

**Disclosure of Interest:**

None Declared

